# Potential Roles of Inflammation on Post-Traumatic Osteoarthritis of the Ankle

**DOI:** 10.3390/ijms25115903

**Published:** 2024-05-28

**Authors:** Pawee Chalidapong, Tanawat Vaseenon, Nipon Chattipakorn, Siriporn C. Chattipakorn

**Affiliations:** 1 Department of Orthopedics, Faculty of Medicine, Chiang Mai University, Chiang Mai 50200, Thailand; purepawee@gmail.com (P.C.); tvaseenon@yahoo.com (T.V.); 2 Neurophysiology Unit, Cardiac Electrophysiology Research and Training Center, Faculty of Medicine, Chiang Mai University, Chiang Mai 50200, Thailand; nchattip@gmail.com; 3 Center of Excellence in Cardiac Electrophysiology Research, Chiang Mai University, Chiang Mai 50200, Thailand; 4 Cardiac Electrophysiology Unit, Department of Physiology, Chiang Mai University, Chiang Mai 50200, Thailand; 5 Department of Oral Biology and Diagnostic Sciences, Faculty of Dentistry, Chiang Mai University, Chiang Mai 50200, Thailand

**Keywords:** post-traumatic osteoarthritis, pilon fracture, intra-articular ankle fracture, inflammation, arthritis

## Abstract

Post-traumatic osteoarthritis of the ankle (PTOA) is frequently observed following a debilitating consequence of intra-articular ankle fractures. Numerous risk factors contribute to the pathogenesis of PTOA, including articular incongruity, joint malalignment, and concomitant soft tissue damage. Despite attempts to restore joint anatomy and manage soft tissues to avoid long-term complications after intra-articular ankle fractures, the incidence of PTOA remains markedly elevated. Inflammatory processes triggered by intra-articular ankle fractures have emerged as potential instigators that expedite the progression of PTOA. Injury to the articular cartilage and subchondral bone may lead to the release of inflammatory mediators, which can contribute to cartilage degradation and bone resorption. This study provides a narrative review on the current knowledge concerning the association between inflammation and the development of PTOA following intra-articular ankle fractures. We also discuss novel therapeutic agents that target inflammatory pathways to impede the progression of post-traumatic osteoarthritis after intra-articular ankle fractures. These medication and interventions were summarized within this review article.

## 1. Introduction

Intra-articular ankle fracture is one of the common fractures found in patients with traumatic injuries and may lead to the development of post-traumatic osteoarthritis (PTOA) [1]. The incidence of PTOA is 50% within the first four years and 75% within the first 5–12 years after the intra-articular ankle fracture [1]. Together with ankle fracture, joint instability, joint malalignment, and articular incongruity, it could accelerate the progression of PTOA, which has been supported by few studies with finite element analysis [2,3]. Those studies that evaluated contact stress in cadaver specimens reported an increase in contact stress of up to 300% when there was a step-off of more than 2 mm between the articular surfaces. This step-off could cause an anatomical shift in the loading pattern of the joint surface, which may lead to progression of osteoarthritis [2,3].

PTOA is the most frequent chronic arthritis of the ankle. The treatment strategies primarily focus on reducing the incidence and severity of PTOA following pilon and rotational malleolar fractures. Both pilon and rotational bimalleolar fractures can lead to the development of PTOA. However, the intra-articular involvement in a pilon fracture poses a greater chance for the development of PTOA than bimalleolar fracture. Although the restoration of articular congruity has been performed, the functional long-term outcomes after surgery are still poor, and the incidence of PTOA has remained high following pilon fracture [4,5] considering calcaneus fractures [6] and talus fractures [7]. Inflammation is a possible contributor to the pathogenesis of PTOA, since it causes a decline in an articular surface, leading to joint deterioration [8]. Interleukins (ILs) are crucial factors in the inflammatory process and are involved in the onset of PTOA. Serum C-reactive protein (CRP) and erythrocyte sedimentation rate (ESR) are inflammatory biomarkers that have been demonstrated to be elevated in patients with PTOA [9]. The duration of elevated levels of ESR and CRP after surgery has not been mentioned in the previous studies. However, most patients with ankle arthritis have intermittent symptoms. Those two inflammatory biomarkers may exhibit a rise-and-fall pattern according to the inflammatory responses independent of post-traumatic osteoarthritis. However, both are non-specific biomarkers.

This review aimed to provide an overview of the potential roles of inflammation in the pathogenesis of PTOA. Particularly, this review focused on the roles of inflammatory mediators, on joint damage following ankle fractures, and on the potential biomarkers to predict the development of PTOA following an intra-articular ankle fracture. We also summarized and discussed current treatments and the possible directions of further research in this field.

**Methods**: We identified the literature for this review article by conducting systematic searches of PubMed for articles published from 2000 until 2023. The search strategies included the keywords ‘post-traumatic’ or ‘traumatic’, ‘ankle arthritis’ or ‘PTOA’, ‘traumatic’ or ‘posttraumatic’ and ‘biomarker’ or ‘cytokines’, which identified 43 publications. The criteria for selection were original research articles written in English published between 2000 and 2023.

By gaining a better understanding of the inflammatory processes involved in the development of PTOA, it is possible to identify new therapeutic targets to prevent the progression of PTOA.

## 2. Post-Traumatic Arthritis of Ankle (PTOA) after Intra-Articular Ankle Fractures

Although the precise mechanism of PTOA development following intra-articular ankle fractures is still unknown, it is recognized that mechanical factors can exacerbate the progression of PTOA [2,3]. The initial trauma could cause cartilage damage. Injury to the articular cartilage and subchondral bone can also lead to the release of inflammatory mediators, which can further contribute to cartilage degradation and bone resorption [10,11,12].

The mechanical alteration of joints following fracture are the straightforward risk factor. Alterations in joint mechanics and increased joint loading occur following the malalignment and can subsequently lead to cartilage degradation and the progression of PTOA. Another risk factor is joint instability [13]. Ligamentous injuries that occur simultaneously with either intra-articular or rotational ankle fracture can cause joint instability, which then leads to a shift in joint loading pattern and subsequently the progression of PTOA [4,5]. Despite achieving a perfect reduction in the articular surface, limited improvement in the functional long-term outcomes, and the occurrence of PTOA, are still reported with high incidence with 39% of patients with tibial plafond fracture [4]. Inflammation is one of the emerging factors that may play a role in the development of post-traumatic osteoarthritis following an ankle fracture [4,5,14].

Pro-inflammatory cytokines, including IL-1, IL-6, IL-8, TNF-a, and PGE2, in ankle synovial fluid have been identified as biomarkers that are associated with an increased risk of developing PTOA following ankle fracture [14,15]. These cytokines can trigger the metabolic responses in the joint, including the recruitment of macrophage inflammatory proteins (MIP), monocyte chemoattractant proteins (MCP), and the promotion of articular cartilage degradation via matrix metalloproteinases (MMP) [13,15,16]. In contrast, anti-inflammatory factors such as IL-4, IL-10, and IL-1 receptor antagonists (IL-1RA) may have a protective effect against the development of PTOA by reducing inflammation in the early post-injury period. Few studies have been conducted to assess the benefits of targeting pro-inflammatory cytokines following joint injury. Furman BD, et al. conducted a study using C57BL/6 mice in 2014 [17]. It was found that intra-articular IL-1RA could significantly reduce the cartilage degeneration and synovial inflammation at 8 weeks post-injury. However, this study was conducted on a mice knee joint, not an ankle joint [17]. Another study was conducted on the effect of IL-1RA on reducing the production of proinflammatory biomarkers including IL-8, MMP-3,10, and CTX-II from the cartilage disc from 0 to 2 days after fracture. This could have an antagonist effect towards inflammation [15,18]. However, more clinical studies may be needed in the future to gain more understanding on this topic.

## 3. Evidence of Inflammatory Responses after Intra-Articular Ankle Fractures

### 3.1. Reports from Clinical Studies

The inflammatory responses that occur after the intra-articular ankle fracture are a critical component of the healing process; however, excessive inflammation can lead to the destruction of cartilage and the development of PTOA.

A previous clinical study reported from the histological findings that a marked increase in scores of synovitis severity (including synovial lining layer enlargement, cell density and inflammatory infiltration with a total maximal synovitis score of 9) in the joints of intra-articular ankle fracture was shown when compared with normal ones [19]. Furthermore, the synovium of the intra-articular ankle fractures exhibited a higher number of CD68+ macrophages. These indicated the inflammatory responses in joints after intra-articular ankle fractures [19]. Several studies also demonstrated that the synovial fluid concentrations of pro-inflammatory cytokines such as IL-1β, IL-6,8, TNF-*α*, IFN-γ, prostaglandin E2 and degradative enzymes such as MMP-1, 2, 3, 8, 9 and 13 in injured intra-articular ankles were significantly higher when compared with those of uninjured ankles [11,19]. An increase in inflammatory responses confirms the persistence of joint inflammation after injury. Surprisingly, Pham TM et al. also found that the concentration of anti-inflammatory cytokines, such as interleukin-1 receptor antagonist (IL-1RA), IL-4 and IL-10 was also increased in the joints of injured ankles [18].

In addition, vascular endothelial growth factor (VEGF), which is one of the anabolic chemokines, was elevated in joints 24 h after an intra-articular ankle fracture [12,19,20,21]. This phenomenon could be due to (1) increased VEGF as the compensated mechanism to neutralize inflammation within the joint, and (2) the upregulation of VEGF to promote healing. Several chemokines were also elevated in the synovial fluid after the intra-articular ankle fractures, including macrophage-derived cytokine (MDC), interferon-induced protein-10 (IP-10), thymus activation-regulated chemokine (TARC), macrophage inflammatory protein (MIP-1b), eotaxin, and monocyte chemoattractant protein (MCP-1, MCP-4) [19]. A previous clinical study reported that synovial fluid from intra-articular ankle fractures contained significantly more heme products than healthy ones, while the amount of sGAG and CTX-II, which are cartilage-degradative products, were not different among the two groups [11].

Furthermore, Adams SB and colleagues reported the temporal fluctuation of inflammatory mediators in ankle joints following intra-articular ankle fractures. The authors found that the rapid surge and decline in levels of pro-inflammatory cytokines including IL-1β and MMP-9 were found in the first two days following injury [21]. The peak sustained levels of IL-10 and IL-4 over the first nine days following injury were accompanied by a decrease in IL-1β following the first two days. Chondrocytes are thought to produce both IL-10 and IL-4, which are believed to protect cartilage against the pro-inflammatory pathways of IL-1β and TNF-α [21]. A surge in MMP and products of cartilage (CTX-II) was found 10 days following the fracture. The highest levels of CTX-II appeared in synovial fluid sampled later than two days post-fracture. These findings suggested that the rise in mediators of cartilage catabolism in the three-to-nine days may start the destruction of joints. Furthermore, MMPs persist beyond ten days, suggesting that MMPs can continue to produce destructive effects on articular cartilages [21].

The role of activating complements after intra-articular ankle fractures were also mentioned as one factor of PTOA development. Hagen Schmal et al. found that during the first six days following fracture, levels of aggrecan and C5b-9 in the synovial fluid increased, which suggested the degradation of the extracellular matrix (ECM) of the cartilage and the accumulation of by-products of complement activation [9]. It has been suggested that C5a may be a connection between complement activation and the function of osteoblasts and osteoclasts. C5a is known to have chemotactic effects on both osteoblasts and osteoclasts. Mesenchymal stem cells were also thought to be involved in fracture healing, and they have expressed receptors for both C5a and TNF-*α* [9].

Not only complement factors, but also b-FGF and IGF-1, increased in ankles following fractures, compared to osteochondral lesion of talus. Both proteins play a significant role in cartilage metabolism. It is known that osteoarthritic progression is associated with the upregulation of b-FGF, which may also be expected in posttraumatic degeneration [9].

Furthermore, the intra-articular ankle fractures showed elevated levels of many lipid metabolites, including most long-chain fatty acids and polyunsaturated fatty acids (PUFAs), suggesting the possible involvement of a phospholipase A2 (PLA2) enzyme [22]. However, most metabolites reversed to normal levels at six months following injury. These metabolites may play a role in PTOA progression. However, the process is still unclear [22].

All these findings are summarized in Table 1.

### 3.2. Reports from In Vivo Studies

Not only have clinical studies been conducted, but also in vivo studies to investigate the inflammatory responses following pilon fracture. Impaired lubrications are associated with the development of arthritis. The lubricin acts as a boundary lubricant, while hyaluronic acid provides synovial fluid with viscoelastic properties. A study conducted by Bridgette T. Peal et al. in 2020 was performed to assess the temporal fluctuation of lubricin and hyaluronan level in equine ankle, wrist, and knee joints. Synovial fluid was collected from clinically healthy, high motion equine joints, talar cartilage with impact injury and joints with osteochondral fragmentation. Lubricin and HA concentrations were then measured with ELISA and analyzed with linear regression models [31]. The study showed that lubricin concentrations increased post-injury, while a decline in hyaluronic acid concentration was found in the osteoarthritis model [31]. This elevation in lubricin could be explained by the compensation for the loss of hyaluronic acid post-injury [31]. However, only the comparative study in 1985 investigated lubricin levels of ankle, MCP, and knee of bovine in comparison with the level of human knee synovial fluid [32]. Lubricin levels in human ankle synovial fluid under physiological conditions have not been mentioned in previous studies.

## 4. Evidence of Inflammatory Responses in Post-Traumatic Osteoarthritis of Ankle (PTOA)

### 4.1. Reports from Clinical Studies

Inflammation plays a role in accelerating the development of PTOA. The potential use of inflammatory cytokines and metabolites in synovial fluid as biomarkers of this degenerative process is still questionable. The studies showed that patients with PTOA had significantly higher concentrations of pro-inflammatory cytokines, including IL-6, IL-8, TNF- α, MMP-3, and monocyte chemoattractant protein (MCP-1) in their synovial fluid compared to control patients [11,19,33]. The increased levels of cytokines and metabolites in synovial fluid may indicate persistent joint inflammation. However, a higher level of anti-inflammatory cytokines, including IL-10 and interleukin-1 receptor antagonist (IL1-RA), which binds to the same receptor as IL-1, did not activate the transcription of pro-inflammatory genes [11]. These findings in PTOA might be explained by the ongoing process of remodeling, which requires both the production and destruction of the chondrocyte and extracellular matrix [19,23,33,34]. In addition, San Giovanni et al. found a positive correlation between the severity of ankle articular cartilage degeneration from intra-operative arthroscopic findings and the levels of IL-6, MCP-1, and fibronectin-aggrecan complex [33]. However, the synovial fluid concentration of IL-1β and MMP-13 showed no significance between PTOA and a healthy ankle [34].

Aggrecan is a large proteoglycan that is specific to cartilage and is an important component of the extracellular matrix (ECM) [34]. When cartilage and matrix break down, aggrecan molecules are released into the joint space. An increase in aggrecan levels in the joint is expected during osteoarthritis with loss of cartilage. Hagen Schmal and colleagues also found that an increase in synovial fluid aggrecan correlated with not only the Kellgren–Lawrence radiographic score, but also with the functional outcomes of PTOA patients [9,34]. Bone morphogenic protein (BMP) is another notable biomarker [34]. BMP-7 was associated with PTOA progression, while high levels of BMP-2 in synovial fluid were associated with good functional outcomes and lower radiographic progression of osteoarthritis [34].

Ghrelin is a 28-amino-acid polypeptide that promotes the release of growth hormone [35]. Liu et al. conducted an in vitro study that showed that ghrelin could impede the expression of matrix metalloproteinase-3 (MMP-3) and ADAMTS5 mediated by interleukin-1β [36]. Ghrelin also prevents the degradation of aggrecan and type II collagen in human chondrocytes [35,36]. The attenuated synovial fluid level of ghrelin concentrations was correlated with the disease severity of ankle PTOA both clinically (indicated by VAS and AOFAS ankle-hindfoot scores) and radiographically (indicated by modified Kellgren–Lawrence score) [35]. A previous study also demonstrated that ghrelin in synovial fluid could have a protective effect on ankle osteoarthritic progression [35,36].

The alterations of biomarkers in PTOA from clinical studies are summarized in Table 2.

### 4.2. Reports from In Vitro Studies

Several in vitro studies were conducted to understand the biomolecular pathogenesis of PTOA [38,39,40]. The upregulation of CXCL10 gene expression was reported in the serum of patients with inflammatory arthritis [38]. Cartilage collected from patients with end-stage osteoarthritis, post-traumatic ankle fracture, and normal patients were also analyzed in that study. The authors demonstrated that a significant increase in CXCL10 expression was found in cartilage after ankle fracture, while a slight increase in CXCL10 expression was detected in OA cartilage. The authors also reported that CXCL10 reduced IL-1α stimulated total MMP and sGAG release [38]. These findings suggested that exogenous CXCL10 may indirectly have anti-inflammatory effects, resulting in preventing the development of PTOA following ankle fracture.

The cytoplasmic polyadenylation element-binding protein1 (CPEB1) gene expression is another interesting topic associated with PTOA. A previous study from Lei Li et al. indicated that CPEB1 was upregulated in articular cartilage of PTOA, and its expression was positively correlated with disease severity [39].

The results of in vitro studies regarding the alterations of biomarkers in PTOA are illustrated in Table 3.

## 5. Evidence of Inflammatory Responses in the Development of Post-Traumatic Arthritis (PTOA) after an Intra-Articular Ankle Fracture

### 5.1. Reports from Clinical Studies

Several studies found that acute intra-articular ankle fractures are associated with a notable increase in the concentration of pro-inflammatory cytokines, including IL-1β, IL-6, IL-8, IL-10, and TNF-α, and MMPs when compared to healthy ankles [41]. Some biomarkers, such as IL-6, IL-8, MMP-1, MMP-2, and MMP-3 remained elevated for at least six months after the fracture [41].

Researchers have also investigated the relationship between a rise in biomarker concentrations in synovial fluid and functional outcomes of PTOA. A previous study showed that higher levels of IL-2 in synovial fluid were associated with worse functional outcomes at 12 months following the fracture. Poor radiographic findings of osteoarthritis by Kellgren–Lawrence scores were linked to elevated concentrations of IL-6 and INF-γ [41]. Interestingly, elevated concentrations of IL-1β after injury were positively correlated with AOFAS score at 12 months after surgery although IL-1β is a pro-inflammatory cytokine that theoretically should harm AOFAS. Therefore, the synovial inflammatory response following an intra-articular ankle fracture may be a naturally repairing phenomenon that has a limited short-term impact on functional outcomes [41]. However, it may be necessary to conduct longer follow-up studies to confirm potential association between these cytokines and joint cartilage.

The profiles of amino acids in synovial fluid following intra-articular ankle fractures and their association with PTOA were also studied. Although there was no significant difference found, a few metabolites, including GSSG, cysteine-glutathione disulfide, C-glycosyltryptophan, glutamate, DSGEGDFXAEGGGVR*, and HWESASXX, had average fold changes over 2.1 [42]. These findings suggested that certain changes in glutathione, tryptophan, and glutamate metabolism may be chronic, and those alterations played a role in the long-term pathology of PTOA [42]. Nonetheless, further investigations are needed to gain an understanding of the roles of amino acids in the onset of PTOA.

The role of CXCL10 protein on the development of PTOA following an intra-articular fracture was also investigated in clinical studies. CXCL10 expression was significantly higher in the synovium and cartilage tissue of patients with articular fractures when compared to healthy controls [38]. In addition, the level of CXCL10 was higher in patients who developed PTOA when compared to that of those who did not [38]. That study also examined the effect of exogenous CXCL10 on chondrocyte catabolism in an in vitro study and discovered that CXCL10 may play a role in modulating inflammation by reducing the production of MMPs [38]. Those findings suggest that although CXCL10 does not directly induce cartilage catabolism, it may have a role in chondrocyte homeostasis and inflammation modulation.

In the comparative analysis of clinical studies among three groups, including intra-articular ankle fractures, PTOA, and PTOA following ankle fractures, the authors found that multiple pro-inflammatory and degradative biomarkers were elevated in the synovial fluid (8, 9, 15, 17, 32, 36–38). However, some biomarkers increased in all three groups, including IL-1β, IL-6, and MMP-3 (8, 9, 15, 17, 32, 36–38), suggesting that these biomarkers could be potential targets for therapeutic interventions for the progression of PTOA.

The alterations in biomarkers in PTOA following the intra-articular ankle fractures from clinical studies are illustrated in Table 4.

### 5.2. Reports from In Vivo Studies

In addition to clinical studies, the researchers established a rat model for post-traumatic osteoarthritis (PTOA) induced by a malleolar fracture with or without reduction [43]. Those rats were then monitored for 12 weeks to evaluate the development of PTOA [43]. This model was developed to imitate the characteristics of human PTOA, making it easier to study the disease and explore potential therapeutic interventions. A higher level of MMP-13 was found in the experimental group by ELISA analysis and fluorescent molecular tomography, suggesting the role of inflammation in the development of PTOA after the malleolar fracture [43].

The results of alterations in biomarkers in in vivo studies of PTOA following intra-articular ankle fractures are illustrated in Table 5.

## 6. Possible Therapeutic Interventions to Inhibit the Development of Post-Traumatic Osteoarthritis of Ankle (PTOA): Reports from Clinical Studies and In Vitro Studies

The possible therapeutic approach of PTOA is to target the inflammatory responses that occur following an ankle injury. The details of therapeutic interventions in previous studies are summarized in the following paragraph.

### 6.1. A Single Intra-Articular Injection of Autogenous Leukocyte-Reduced Platelet-Rich Plasma

This may be beneficial in acute closed pilon fracture during the early phase within 24 h after injury, as it has anti-inflammatory, anti-degradative, and anabolic effects. A prospective randomized double-blind clinical trial by Bradford P. Zitsch et al. was performed in eleven injured patients: six of them were treated with PRP, while the rest were injected with saline at the time of external fixator placement. It was found that PRP-treated synovial fluid contained significantly higher levels of PDGF-AA and lower levels of MMP-3, MMP-9, IL-1β, IL-6, IL-8 and PGE2 compared to the saline-treated group [20]. The limitations of the study are that the authors focused on the assessment of biomarkers in synovial fluid without functional and radiographic assessment of PTOA [20].

Evidence of inflammatory responses after intra-articular ankle fractures from clinical studies with interventions are shown in Table 6.

### 6.2. Resolvins (RVs)

Represent a class of small bioactive compounds that have an anti-inflammatory action [26]. RVs are endogenously derived from the omega-3 polyunsaturated fatty acid, docosahexaenoic acid (DHA) [26]. Resolvin D1 (RvD1) is known for its robust anti-inflammatory and erythropoietic properties [26]. In vitro studies with LPS-induced MG-63 cells from Dan Cao et al. were conducted in 2018. Pre-treatment of RvD1 dose-dependently reversed the LPS-induced upregulation of IL-1 β, IL-6, TNF-α, and COX-2 mRNA and protein levels, and decreased expression of the p-p38, NF-κB (50), and NLRP3 inflammasome, including NLRP3, ASC, and cleaved caspase-1/caspase-1 induced by LPS treatment in MG-63 cells. RvD1 demonstrated efficacy in the treatment of inflammation by repressing nuclear factor-kappa B (NF-κB) signaling pathways as well as by suppressing the expression of the nucleotide-binding oligomerization domain-like receptor family pyrin domain-containing 3 (NLRP3) inflammasome in MG-63 cells [26]. These various mechanisms contribute to attenuate inflammatory processes. RvD1 shows potential as a pharmacological agent to treat the progression of PTOA [26].

### 6.3. N-methyl Pyrrolidone (NMP)

This small bioactive compound showed action in promoting bone growth and inhibiting osteoclast differentiation [21]. The in vitro studies with BK-induced MG-63 cells from Jun Bian, et al. were conducted in 2018. Measures of 5 mM and 10 mM NMP could both significantly reverse the BK-induced increase in COX-2 and iNOS. A measure of 10 mM NMP could also significantly reverse the increase in the level of TNF-α, IL-1β and IL-6 mRNA. However, 5 mM NMP could only reverse the rise in the level of IL-6 mRNA significantly. NMP also markedly inhibited the BK-induced overactivation of JNK and p38, which in turns may suppress inflammation in the joint via these pathways [27].

### 6.4. miR-146a

Is involved in the development and progression of several diseases, including arthritis, tumors, and coronary heart disease [28]. In addition, the researcher has revealed that miR-146a plays a significant role in the management of inflammation and oxidative stress. The in vitro studies with BK-induced MG-63 cells from Haijun Mao et al. were conducted in 2018. According to this study, miR-146a protected against inflammation following ankle fracture by inhibiting the TRAF6/NF-κB pathway and reducing oxidative stress. It also reduced the expression of pro-inflammatory biomarkers, including TNF-α, IL-1β and IL-6, in BK-induced MG-63 cells. Therefore, miR-146a might promote ankle healing and thus may be one of the potential therapeutic targets for this scenario [28].

### 6.5. Sinomenine (SIN)

Is an active alkaloid derived from *Sinomenium acutum*, a plant known for its therapeutic properties [29]. SIN exhibits analgesic, anti-arthritic, anti-inflammatory, and immunosuppressive characteristics [29]. The in vitro studies with BK-induced MG-63 cells from Jie Shen et al. were conducted in 2018. According to the study, SIN pretreatment remarkably reduced p-p38 and p-NF-kB protein expression. It also increased the Nrf2, HO-1, and NQO-1 protein and mRNA expression in a dose-dependent manner. Therefore, SIN could serve as a potential therapeutic agent to slow the progression of PTOA by modulating oxidative stress and inflammation through the MAPK, NF-kB, and Nrf2 signaling pathways [29].

### 6.6. Propofol

Propofol, on the other hand, shows anti-inflammatory effects [24] according to the study from Ping Zhou et al. in 2018, which investigated the effect of propofol on the inflammatory response in BK-induced MG-63 cells. Propofol was shown to significantly inhibit a BK-induced increase in TNF-a, IL-1b, and IL-6 in BK-induced MG-63 cells. It also significantly inhibited BK-induced activation of inflammation via p38, MAPK, and NF-kB pathways in a dose-dependent (1, 5, and 10 μg/mL) manner. Its suppression of NLRP3 and cyclooxygenase-2 (COX-2) expression may help protect the ankle against the progression of PTOA [24].

### 6.7. IL-1 Receptor Antagonist (IL-1RA) and Doxycycline

According to the in vitro study from Nicholas B. Allen et al. in 2022, which obtained synovial fluid from 54 intra-articular fractures cultured with cartilage discs from the dome of fresh allograft human tali, these two agents may have potential effects in reducing PTOA by diminishing the concentrations of pro-inflammatory cytokines and cartilage degradative products, such as IL-8, matrix metalloproteinase-3 (MMP-3), MMP-10, and C-terminal telopeptide of type II collagen (CTX-II), within the synovial fluid [44]. Consequently, the attenuation of inflammatory mediators can contribute to the mitigation of PTOA [44].

### 6.8. Surfactant P188

P188 in a single dose had beneficial effects on chondrocyte survival, apoptosis inhibition, and the maintenance of cartilage integrity [40]. That study was conducted by Sarbottam Baiai et al. in 2010, using ankle cartilage obtained from human tissue donors. Studied pathways were activated 1 h after impaction with the peak of activity. P188 was shown to completely attenuate phosphorylation of STAT1, ATF-2 and inhibit p38, Stat3, JNK, ERK and GSK3. According to the study, P188 was found to stabilize cellular membranes, inhibit the activation of p38, glycogen synthase kinase-3 linked to apoptosis, as well as the inflammation associated with IL-6 signaling [40]. Those findings suggested that P188, by itself or co-administered with growth factors, could be a potential means of preventing PTOA.

All possible therapeutic interventions of PTOA from in vitro studies are shown in Table 7.

Hyaluronic acid (HA) is one of the known substances used in treating joint arthritis, including ankle arthritis. There are some studies on the effect of intra-articular HA injections in ankle arthritis. A study by C. Jantzen et al. in 2020 also indicated that a single intra-articular injection of HA has been insufficient to produce a clinically relevant response at 6 months [45]. However, the limitation of that study was the small sample size. In addition, a systematic review in 2018 on the effect of intra-articular ankle injections for treating symptoms of ankle arthritis revealed a significant improvement in scores on the ankle osteoarthritis scale with HA compared to saline injections at 6 months [46]. However, most studies of HA as a joint health supplement were conducted on ankle arthritis rather than on PTOA.

Methylsulfonylmethane (MSM) is a dietary supplement with anti-inflammatory effects; however, it is mostly used for knee joint arthritis. A prospective, randomized, double-blind controlled clinical trial was conducted in 2023 to assess the efficacy of MSM in treating patients with knee osteoarthritis, and the results showed an improvement in pain and physical function [47]. Despite those findings, no current study has examined the role of MSM in patients with ankle arthritis or PTOA.

Although several possible therapeutic interventions can inhibit the development of post-traumatic osteoarthritis of the ankle, biomechanical factors, which can be managed with good osteosynthesis techniques, are still straightforwardly important. Further studies are still needed to determine the most effective approaches and optimal timing of interventions to prevent long-term joint damage following ankle injury.

## 7. Conclusions

The development of PTOA after intra-articular ankle fractures is influenced by multiple factors, including post-injury inflammation, which has been identified as a key contributor to accelerating the degenerative process. Both pro-inflammatory and degradative biomarkers, as well as anti-inflammatory and anabolic biomarkers, are involved in the process of cartilage degeneration. Changes in the concentration of chemokines and metabolites in the synovial fluid have also been observed after intra-articular ankle fractures.

**Figure 1:**
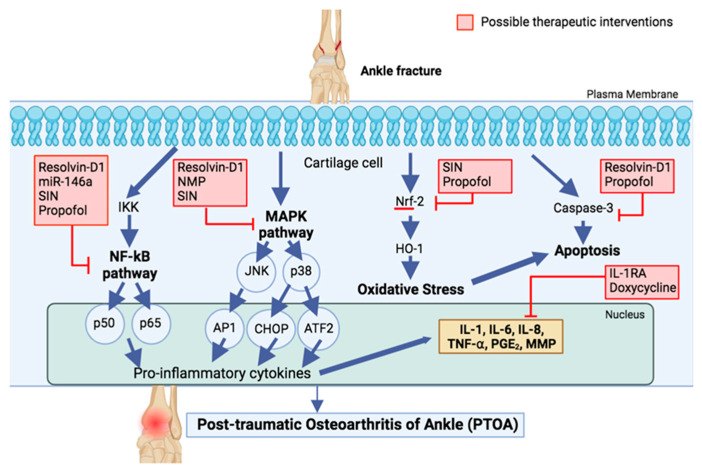
Possible Underlying Mechanisms of Inflammation Leading to Development of PTOA After Ankle Fracture, and Therapeutic Approaches. The intricate signaling cascades are implicated in the initiation and progression of inflammatory responses after ankle fractures, along with plausible therapeutic agents that can be employed to attenuate inflammation. Resolvin D1 (RvD1) exhibits promising inhibitory properties towards Nf-kB, MAPK, and apoptotic pathways by impeding caspase-1 and caspase-3 activation. Similarly, SIN impedes Nf-kB, MAPK pathways, and oxidative stress through the activation of Nrf-2. NMP selectively hampers the MAPK pathway, while miR-146a specifically targets the Nf-kB pathway. Propofol manifests potential inhibitory effects on Nf-kB, oxidative stress, and apoptotic pathways. Notably, IL-1RA and doxycycline represent alternative inhibitors that have the potential to mitigate the levels of pro-inflammatory cytokines.

Several therapeutic interventions, including leukocyte-reduced platelet-rich plasma, interleukin-1 receptor antagonist (IL-1RA), and Resolvin-D1, have been studied to identify effective treatments against the progression of PTOA. However, it is important to note that these interventions cannot replace the importance of achieving perfect anatomical reduction of the articular surface and fracture stabilization in the initial treatment. All findings are summarized in Figure 1.

Research on biological mediators is still in its early stages, and further studies are needed to identify the optimal preventive treatment for PTOA. Overall, a better understanding of the underlying mechanisms of PTOA and continued research efforts are necessary to develop effective therapies for this condition.

## Figures and Tables

**Table 1:** Evidence of Inflammatory Responses After Intra-Articular Ankle Fractures: Reports from Clinical Studies.

Study Model (N: M/F)	Age (yrs)	Major Findings					Interpretation	Ref
		Inflammatory Biomarkers			Oxidative Process	Histologic Findings		
		Pro-	Anti-	Others				
Prospective Randomized Double-blind Controlled trial (11:6/5)	39 ±12	↑ MCP-1 ↑ IL-1β ↑ IL-6 ↑ IL-8 ↑ PGE2 ↑MMP-1,3,8,9	↑ IL-1RA	↑ VEGF	-	-	Pro-inflammatory, anti-inflammatory, degradative, and anabolic biomarkers were elevated 24 h post-acute fracture period.	[20]
Prospective Cohort (24:4/2)	51.8 ± 30.1	↑ IFN-γ ↑ IL-1β ↑ IL-6 ↑ IL-8 ↑ IL-12p70 ↑ TNF-α ↑ IFN-γ	↑ IL-10	Chemokines ↑ Eotaxin ↑ Eotaxin-3, ↑ MCP-1,4 ↑ MDC ↑ MIP-1β ↑ TARC ↑ MCP-1	-	Fracture: ↑Synovitis score ↑CD68+ macrophage	Multiple cytokines and chemokines in synovial fluids increased after ankle fracture.	[19]
Cross-section (47:22/25)	42± 14.4	↑ IL-1β ↑ IL-2 ↑ IL-6 ↑ IL-8 ↑ IL-12p70 ↑ TNF ↑ IFN-γ ↑ MMP-1,3,9	↑ IL-1RA ↑ IL-4 ↑ IL-10 ↑ IL-13	Cartilage degradative products ↑ ACG ↑ CTX-2 Metabolic mediators ↑ TGF-β1 ↑ TGF-β2	-	-	Elevated level of multiple cytokines and mediators was observed after acute intra-articular ankle fractures.	[18]
Cross-section (16: 10/6)	35.94 (24–48)	↑ IL-2 ↑ IL-6 ↑ IL-17 ↔ IFN-γ	↑ IL-10 ↔ TGF-β1	-	-	Synovium ↑ thin ↓thick collagen fiber Chondral tissue: ↑ Cellularity ↑ Articular irregularity ↑Discontinuity ↑ Disarray ↑ PTG ↑ Collagen type 1,3,5	Pro- and anti-inflammatory levels were increased with histologic changes of disarray collagen fibers, increased cellularity, and deposits of PTG after intra-articular ankle fracture.	[10]
Cross-section (21:13/8)	42 (29–63)	↑ IL-1β, 6, 8 ↑ TNF-α ↑ MMP-1, 2, 3, 9, 10	↑ IL-10	↑ GM-CSF ↑ bilirubin/ biliverdin ↔ sGAG ↔ CTX II	-	-	Pro-inflammatory and cartilage degradative products were significantly elevated after intra-articular ankle fracture.	[12]
Cross-section (21:13/8)	42 (20–63)	At6 mo persist: ↑ IL-6 ↑ IL-8 ↑ MMP-1,2,3	-	At 6 mo persist: ↔ sGAG ↔ CTX II	-	-	Pro-inflammatory cytokines levels were persistently increased 6 months after fracture.	[23]
Prospective Cohort (20:8/12)	42 (20–63)	↑ MMP-1,2,9,10 ↑ IL-6 ↑TNF-α	↑ IL-10	↑ Free FA ↑ PUFA ↑ 2-hydroxyl FA ↑sphingomyelins ↑ lysolipids ↑bilirubin/ billiverdin 6 months post-Sx (Reversal) ↓ FA ↓sphingomyelins	-	-	Increasing levels of lipid biomarkers and metabolites were found after ankle fracture, suggesting the possible involvement of phospholipase A2 in arthritic progression.	[22]
Cross-section (30:15/15)	7± 1.1	↑ TNF-α ↑ IL-1β ↑ IL-6	-	-	-	-	Pro-inflammatory cytokines were increased in children with ankle fractures.	[24]
Prospective Cohort (54:24/30)	47 (18–74)	Initial (0–2 d): ↑↑ IL-1β ↑ IL-6 ↑ IL-12p70 ↑ MMP-9 At 3–9 d: ↑ IL-1β ↑ IL-6 ↑ IL-12p70 ↑↑ MMP1 ↑↑ MMP2 ↑↑ MMP3 ↑↑ MMP10 At >=10 d: ↓ IL-1β ↓ IL-6 ↓ IL-12p70 ↔ INF-γ ↔ IL-2, 8, 13 ↔ TNF-α	Initial (0–2 d): ↑ IL-4 ↑ IL-10 >=10 d: ↓ IL-4 ↓ IL-10	Initial (0–2 d): ↑ sGAG At 3–9 d: ↑ CTX II ↑↑ Heme At >=10 d: - ↓ sGAG - ↑↑CTX-II	-	-	Temporal fluctuations in biomarker levels as a result of trauma and hemarthrosis needed to be considered in the pathogenesis of PTOA.	[21]
Cross-section (65:13/23)	39.6 (20–60)	Intra-articular fx ↑ IL-1β ↑ IL-6 ↑ IL-8	Intra-articular fx ↑ IL-1RA ↑ IL-10	Intra-articular fx ↑ MMP-1 ↑ MMP-3 ↑ MMP-13		-	Greater rates of PTOA after plafond fractures, with higher levels of pro-inflammatory and degradative products, were found when compared to extra-articular ankle fractures.	[25]
Cross-section (70:35/35)	5.5 ±1.3	Serum level ↑ TNF-α ↑ IL-1β ↑ IL-6	-	-	-	-	Pro-inflammatory cytokines were increased in children with ankle fractures.	[26]
Cross-section (120:60/60)	6.0 ±1.2	Serum level ↑ TNF-α ↑ IL-1β ↑ IL-6	-	-	-	-	Pro-inflammatory cytokines were increased in children with ankle fractures.	[27]
Cross-section (120:60/60)	12–53	Serum level ↑ TNF-α ↑ IL-1β ↑ IL-6	-	-	↓ miR-146a Oxidative process: ↑ MDA ↓ SOD ↓ CAT	-	Elevation of Pro-inflammatory cytokines and oxidative process were found in children with ankle fracture	[28]
Cross-section (120: N/A)	-	Serum level ↑ TNF-α ↑ IL-1β ↑ IL-6	-	-	Oxidative process: ↑ MDA ↓ SOD ↓ CAT	-	Pro-inflammatory cytokines and oxidative processes were higher in children with ankle fractures compared to those with healthy ankles.	[29]
Cross-section (6: 2/4)	42 ± 24	-	-	↑↑CD3 T-cell (↑↑CD4, CD8) ↑ CD19+ B-cell ↑ CD14+ Monocytes	-	-	Prominent immune cell CD4 and CD8 T-cell infiltration were found after intra-articular fracture.	[30]
Cross-section (8:4/4)	38.9 ±20.6	Ankle fracture ↓ IL-1β	-	First 6 days after fracture ↑Aggrecan ↑ C5b-9 Ankle fracture ↑ C3a ↑ C5a ↑ C5b-9 ↑ bFGF ↑ IGF-1	-	-	Elevation of complements, pro-inflammatory, and anabolic factors were reported after ankle fracture, leading to the development of PTOA	[9]

Abbreviations: ACG, accelerator globulin; bFGF, basic fibroblast growth factor; C3a, complement 3a; C5a, complement 5a; C5b-9, complement 5b-9; CTX-II, C-terminal telopeptide-II; FA, fatty acid; GM-CSF, Granulocyte Macrophage Colony Stimulating Factor; IFN- γ, interferon- γ; IGF-1, insulin-like growth factor-1; MMP, matrix metalloproteinase protein; IL, interleukin; MCP-1, Monocyte Chemotactic Protein-1; MDC, macrophage-derived chemokine; MIP-1β, macrophage inflammatory protein-1β; PGE2, prostaglandin E2; TNF-α, tumor necrosis factor- α; TARC, thymus and activation-related chemokine; TGF-β2, transforming growth factor-β2; sGAG, Sulphated Glucosaminoglycans; PTG, Proteoglycans; PUFA, polyunsaturated fatty acid; PLA2, Phospholipase A2; PTOA, post-traumatic osteoarthritis of ankle VEGF, vascular endothelial growth factor.

**Table 2:** Evidence of Inflammatory Responses in Post-Traumatic Arthritis (PTOA): Reports from Clinical Studies.

Model (N: Gender M/F)		Major Findings				Association	Interpretation	Ref.
	Age (yrs.)	Inflammatory Biomarkers		Functional Outcomes	Radiographic Outcomes			
		Synovial Fluid	Serum					
Cross-section (49:31/18)	32.6 ±13.6	↑ Aggrecan ↑ BMP-7 ↓ BMP-2 ↔ MMP-13 ↔ IL-1β	-	↓ AOFAS ↓ FFI	↑ KLS	Poor outcome ↑ Cartilage degradative products ↑ Anabolic biomarkers	Aggrecan and BMP-7 levels were elevated in PTOA with poor functional and radiographic outcomes.	[34]
Cross-section (36:21/15)	45 ± 16	↑ MCP-1 ↑ IL-6 ↑ FAC	-	↑ Intra-op arthroscopic OA severity grading ↑ VAS	-	Poor outcome ↑ Pro-inflammatory cytokines	Pro-inflammatory cytokines and chemokines were elevated in more severe PTOA findings from the arthroscope.	[33]
Cross-section (97:63/34)	48.2 ± 2.4	↑ Ghrelin ↓ TNF-α ↓ MMP-3	↔ Ghrelin	↑ AOFAS ↑ VAS	↓ Modified KLS ↓ Mankin cartilage score	Good outcomes ↑ Ghrelin ↓ Pro-inflammatory cytokines ↓ Degradation	Increased levels of ghrelin in synovial fluid were inversely proportionated with the severity of PTOA.	[36]
Cross-section (40:17/23)	63 ±15	↑ IL-6 ↑ IL-8 ↑ MCP-1 ↑ IL-1Ra ↑ IL-10 ↑ IL-15		↓ ADL ↓ Response to non-operative treatment	↑ Tanaka-Takakura staging	Poor outcomes ↑ Pro-inflammatory cytokines ↑ Chemokines ↑ Anti-inflammatory cytokines	Elevation of pro- and anti-inflammatory cytokines was found in PTOA after ankle fracture.	[11]
Cross-section (126:94/32) 50 RA 25 OA 10 PA 16 PG 9 GA 16 TA	-	↑ IgG-RF in RA > TA ↑ IgM-RF in RA > TA ↑ IgA-RF in RA > TA ↑ MMP-3 in RA > TA ↔ TIMP RA, TA	-	-	↑ KLS	Poor outcome ↑ Immuno-globulin	Rheumatoid factors (including IgA, IgG, and IgM) were significantly higher in RA compared to TA.	[37]

Abbreviations: AOFAS, The American Orthopedic Foot and Ankle Score; BMP, Bone Morphogenetic Proteins; FAC, Fibronectin–Aggrecan complex; FFI-DK, translation and validation of the Danish Foot Function Index; GA, gouty arthritis; KLS, Kellgren–Lawrence score for osteoarthritis; IgG-RF, immunoglobulin G-rheumatoid factor; IgM-RF, immunoglobulin M-rheumatoid factor; IgA-RF, immunoglobulin A-rheumatoid factor; IL, interleukin; MCP-1, Monocyte Chemotactic Protein-1; MMP, matrix metalloproteinase protein; OA, osteoarthritis; OCD, osteochondral defect; PA, psoriatic arthritis; PG, pyogenic arthritis; RA, rheumatoid arthritis; SF, synovial fluid; TIMP, tissue inhibitor of metalloproteinase; TA, traumatic arthritis; VAS, visual analog scale.

**Table 3:** Evidence of Inflammatory Responses in the Development of Post-Traumatic Arthritis (PTOA): Reports from In vitro Studies.

Study Model	Treatment (Dose/Duration)	Major Findings		Interpretation	Ref.
		Inflammatory Responses	Gene Expression		
Chondrocytes	Transfection with c CPEB1 IL-1β + TNF-α (24 h) Overexpressed CPEB1 + IL-1β (24 h)	- ↑ IL-1β ↓ IL-1β ↑ MMP-3,13 ↑ ADAMTS5	↑ CPEB1 ↑ CPEB1 ↑↑ CPEB1	The upregulation of CPEB1 in chondrocytes was observed in PTOA; however, the overexpression of CPEP1 reduced the pro-inflammatory cytokines.	[39]
Human-impacted Tali cell culture impacted with peak activity (1–600 N)	Impact with peak activity (1–600 N) alone P188 (8 mg/mL, 20 min, 1 h, 24 h) p38 inhibitors (20 µM/ 20 min, 1 h, 24 h) p38i + P188 (20 µM; 8 mg/mL/ 20 min, 1 h, 24 h)	IL-6: ↑↑ at 24–48 h ↓ after 48 h ↑ D12–14 - - -	↑ Stat3 ↑ ATF-2 ↓ COL-II ↓ Stat1 ↓ p38 ↓ Stat3 ↓ GSK3 ↓ ERK ↓ JNK ↑ ATF2 ↔ Elk-1 ↔ ATF-2 ↓ p38 ↓ Stat3 ↓ GSK3 ↓ ERK ↔ JNK ↓ apoptosis ↑cell survival ↓ p38 ↓ ERK ↓ JNK	P188 was a potential therapy in the progression of PTOA via inhibition of three main pathways, including p38, mitochondrial apoptosis-related GSK3, and IL-6 inflammation.	[40]
Cartilage collected from mature female pigs 2. Cartilage from patients with end-stage OA underwent total ankle arthroplasty	Exogenous CXCL10 (1, 10, and 100 ng/mL, 72 h) Cartilage + IL-1α (10, 100 pg/mL, 72 h) Exogeneous CXCL10+ Cartilage + IL-1α (10, 100 pg/mL, 72 h)	↓ Total MMP ↓ Aggrecanase ↔ S-GAG ↔ NO ↑ Total MMP ↑ S-GAG ↑ NO ↔ aggrecanase ↓ Total MMP ↓ S-GAG ↔ NO	-	Although exogenous CXCL10 did not induce cartilage catabolism, it was involved in mitigating inflammation and reducing the production of MMP.	[38]

Abbreviations: ADAMTS5, A distegrin and metalloproteinase with thrombospondin motifs 5; ATF2, Activating Transcription Factor-2; CPEB1, cytoplasmic polyadenylation element-binding protein 1; CXCL10, C-X-C motif chemokine ligand 10; Elk-1, transcription factor of Ets domain; ERK, extracellular-signal-regulated kinase; GSK3, glycogen synthase kinase 3; IL, interleukin; JNK, Mitogen-Activated Protein Kinase 8; MMP, matrix metalloproteinase; p38, p38 mitogen-activated protein kinase; NO, nitric oxide; PTOA, post-traumatic osteoarthritis of ankle; S-GAG, sulphated glycosaminoglycans; Stat, Signal Transducer and Activator of Transcription; TNF, tumor necrosis factor.

**Table 4:** Evidence of Inflammatory Responses in the Development of Post-Traumatic Arthritis (PTOA) after Intra-articular Ankle Fracture: Reports from Clinical Studies.

Model (N: Gender M/F)	Time to PTOA after the Intra-Articular Ankle Fracture (mo.)	Age (yrs)	Major Findings					Clinical Outcome		Associated Factors with PTOA Outcome	Interpretation	Ref.
			Inflammatory Cytokines			Gene Expression	Radio- Graph ic Outcome					
			Synovial Fluids									
			Pro-	Anti-	Others							
Prospective cohort (46:18/24)	12	42.7 ±3.6	↑ IL-2 ↑ IL-6 ↑ IFN-γ ↓ IL-1β	↑ IL-4 ↑ TGF-β2		-	↓KLS	↓ AOFAS ↓ EQ5D-5L ↓ FFI-DK		Poor outcomes ↑ Pro-inflammatory cytokines ↑ Anti-inflammatory cytokines	Both pro- and anti-inflammatory cytokines, except IL-1β, were elevated in ankle fracture with poor PTOA changes	[41]
Prospective cohort (19:7/12)	6	42± 22	↑ IL-1 ↑ TNF-α ↑ FGF ↑ TGF-β ↑↑ MMP-1 ↑↑ MMP-9 ↑ MMP-2 ↑ MMP-3 ↑ MMP-10	-	↑ DSGEGDFXAEGGGVR ↑ HWESASXX ↑ GSSG ↑ Cysteine-Glutathione disulfide ↑ Tryptophan metabolites ↑ Glutamate ↑ Aspartate	-	-	-	Poor outcomes ↑ Pro-inflammatory cytokines ↑ Fibrinogen cleavage peptide ↑ Glutathione metabolites ↑ Tryptophan pathways ↑ Glutamate metabolism ↑ Aspartate metabolism		Elevated metabolites associated with glutathione associated with increased MMP-1,9, suggested the possible role of oxidative stress in PTOA.	[42]
Cross-sectional Cadavers (47:26/20) -Articular fracture -PTOA -Normal	-	-	-	-	-	↑↑ CXCL10 after fracture ↑ CXCL10 in OA ↑ CXCL10 in normal + TNFα, IL-1β	-	-	-		CXCL10 level was increased after fracture, PTOA, and inflammation	[38]

Abbreviations: ADL, activities of daily living; AOFAS, The American Orthopedic Foot and Ankle Score; CXCL10, C-X-C motif chemokine ligand 10; EQ5D-5l, EuroQol-5 Dimensions-5 Levels; FFI-DK, translation and validation of the Danish Foot Function Index; IL, interleukin; IFN, interferon; KLS, Kellgren–Lawrence score for osteoarthritis; MMP, matrix- metalloproteinase; TGF-β2, transforming growth factor-β2; TNF-α, tumor necrosis factor-α; VAS, visual analog scale.

**Table 5:** Evidence of Inflammatory Responses in the Development of Post-Traumatic Arthritis after the Intra-articular Ankle Fracture: Reports from In vivo Studies.

Animals	Model of Ankle Fracture	Major Findings			Interpretation	Ref.
		Inflammatory Responses	Gene Expression	Histological Changes Representing PTOA at 8 Weeks after Fracture		
Mice	Articular fracture	↑ Serum CXCL10 after fracture	↑ CXCL10	-	CXCL10 was upregulated in acute fracture.	[38]
Mice	Malleolar articular fracture + dislocation, immediate reduction Malleolar articular fracture	Synovial fluid profile of both groups ↑ MMP-13 ↑ Col-X ↓ Col-II	Synovium both groups ↑ mRNA for MMP-13 ↓ Col2a1 ↓ Acan	↑ Modified OARSI score in both groups ↑ Cartilage fibrillation ↓ Aggrecan ↓ Matrix ↓ Chondrocytes	Both fracture alone and fracture with dislocation led to increased inflammatory responses in synovial fluid, resulting in the development of PTOA as indicated by damage to the articular cartilage of the talus.	[43]

Abbreviations: Acan, aggrecan; Col-II, collagen type II, Col-X, collagen type X; CXCL10, C-X-C motif chemokine ligand 10; fx, fracture; MMP, matrix metalloproteinase; mRNA, messenger RNA; OARSI, Osteoarthritis Cartilage Histopathology Assessment System.

**Table 6:** Evidence of Inflammatory Responses after Intra-Articular Ankle Fractures: Reports from Clinical Studies with Intervention.

Study Model (N: Gender M/F)	Age	Mode of Fracture	Intervention (Dose/Duration/Route)	Major Findings		Interpretation	Ref
				Biomarkers	Functional Outcome		
Prospective Randomized Double-blind Controlled trial (11: 6/5)	39 ± 12	Closed intra-articular fracture (AO/OTA type 43C)	Autogenous Leukocyte-reduced PRP (5 mL/IA)	(1) Pro-inflammatory ↓ IL-1β ↓ IL-6 ↓ IL- 8 ↓ PGE-2 (2) Anabolic agent ↑ PDGF-AA (3) Degradative products ↓ MMP-3 ↓ MMP-9 (4) Anti-inflammatory ↓ IL1-RA ↓ IL-10	-	Single intra-articular leukocyte-reduced PRP had anti-inflammatory, anti-degradative, anabolic effect compared to saline.	[20]

Abbreviations: AO/OTA, AO/Orthopaedics Trauma Association fracture and dislocation classification; IL, interleukin; MMP, matrix metalloproteinase; PGE2, Prostaglandin E2; PDGF-AA, platelet-derived growth factor-AA; PRP, platelet-rich plasma; PTOA, post-traumatic osteoarthritis of the ankle.

**Table 7:** Evidence of Inflammatory Responses after Bone Cell Injury: Reports from In Vitro Studies.

Study Model (Cell Line)	Intervention (Dose/Duration/ Route)	Major Findings					Interpretation	Ref
		Inflammatory Biomarkers	Oxidative Process	Gene Expression	Apoptosis	Cell Viability		
MG-63 cells	LPS (1 μg/mL) LPS + RvD1 (50, 100, 200 nM /2 h)	↑ TNF-α ↑ IL-1β ↑ IL-6 ↑ COX-2 ↓ TNF-α ↓ IL-1β ↓ IL-6	- -	↑ p38 ↑NFKB(p50) ↑ NLRP3 ↑ ASC ↓ LPS-induced proliferation inhibition ↓ p-p38 ↓ p50 ↓ NLRP3 ↓ ASC	↑ cleaved caspase-1 ↑ caspase-1 ↓ cleaved caspase-1 ↓ caspase-1	↓ ↑	RvD1 inhibited inflammation following ankle fracture via MAPK, NF-kB, and NLRP3 inflammasome pathway.	[26]
MG-63 cells	BK induction (1 μM, 24 h) BK + NMP (5 mM, 48 h) BK + NMP (10 mM, 48 h)	↑ TNF-α ↑ IL-1β ↑ IL-6 ↑ COX-2 ↑ iNOS ↔ TNF-α ↔ IL-1β ↓ IL-6 ↓ COX-2 ↓ iNOS ↓ TNF-α ↓ IL-1β ↓ IL-6 ↓ COX-2 ↓ iNOS	- - -	↑ p-ERK ↑ p-JNK ↑ p-p38 - ↔ p-ERK ↓ p-JNK ↓ p-p38	- - -	- - -	NMP inhibited inflammation of the protein kinase pathway after ankle fracture.	[27]
MG-63 cells	BK induction (1 μg/mL, 24 h) BK + miR-146a mimic BK + miR-146a inhibitor	↑ TNF-α ↑ IL-1β ↑ IL-6 ↓TNF-α ↓ IL-1β ↓ IL-6 ↑ TNF-α ↑ IL-1β ↑ IL-6	↑ MDA ↓ SOD ↓ CAT ↓ MDA ↑ SOD ↑ CAT ↑ MDA ↓ SOD ↓ CAT	↑ TRAF6 ↑ p-NF-kB ↑ p-p-65 ↓TRAF6 ↓ p-p-65 ↑ TRAF6 ↑ p-NF-kB ↑ p-p-65	- - -	↓ ↑ -	miR-146a protects against inflammation, and oxidative stress and inhibits TRAF6/NFκB pathway following ankle fracture.	[28]
MG-63 cells	BK induction (1 μg/mL, 24 h) BK + SIN pretreatment (0.25, 0.5, or 1 mM, 2 h)	↑ TNF-α ↑ IL-1β ↑ IL-6 ↓TNF-α ↓ IL-1β ↓ IL-6	↑ MDA ↓ SOD ↓ CAT ↓ MDA ↑ SOD ↑ CAT	↑p-p-38 ↑p-NF-kB (p65) ↓ Nrf2 ↓ HO-1 ↓ NQO-1 ↓ p-p-38 ↓ p-NF-kB (p65) ↑Nrf2 ↑ HO-1 ↑ NQO-1	- -	- -	Sinomenine was one of the potential agents for reducing inflammation and oxidative stress after ankle fracture.	[29]
54/Cross-section - control - SFFH D0-2 - SFFH D3-9 -SFFH D10-14 - with IL-1RA - with Doxy	SFFH D0-2 SFFH D3-9 SFFH D3-9 SFFH D10-14 IL-1RA (20 μg/mL, added to culture) Doxycycline (known MMP inhibitors) (25 μg/mL, added to culture)	↑ IL-8 ↑ MMP-1 ↑ MMP-3 ↑ MMP-10 ↑ CTX-II ↑ sGAG ↓PTG ↓ IL-8 ↓ MMP-3 ↓ MMP-10 ↓ CTXII ↓ IL-8 ↓ MMP-3 ↓ MMP-10 ↓ CTXII	- - - -	- - - -	- - ↔ ↔	↔ - - -	Fracture hematoma in synovial fluid causes cartilage damage through multiple pro-inflammatory cytokines; IL-RA and doxycycline could potentially reduce PTOA by inhibiting these markers.	[44]
MG-63 cells	BK induction (1 μg/mL, 24 h) BK + Propofol (1, 5, 10 μg/mL, added in cell culture)	↑ TNF-α ↑ IL-1β ↑ IL-6 ↓ TNF-α ↓ IL-1β ↓ IL-6	- -	↑ p-p38 ↑ p-p65 ↑ NLRP3 ↑ ASC ↓ p-p38 ↓ p-p65 ↓ NLRP3 ↓ ASC	↑Caspase-1 ↓Caspase-1	- -	Propofol reduces activation of MAPK, NF-kB, and NLRP3 pathways, which makes it beneficial for ankle fracture healing.	[24]

Abbreviations: ASC, apoptosis-associated speck-like protein containing a caspase recruitment domain; BK, human polyomavirus; CAT, Catalase; COX-2, Cyclo-oxygenase 2; RvD1, Resolvin D1; HO-1, heme oxygenase-1; IL, interleukin; iNOS, Inducible Nitric Oxide Synthase; LPS, Lipopolysaccharide; NFkB, nuclear factor kappa-light-chain-enhancer of activated B cells; NLRP3, Cryopyrin; NMP, N-methyl pyrrolidone; MG-63, MG-63 osteoblast-like cells; miR-146a; micro ribonucleic acid 146a; MAPK, mitogen-activated protein kinase; MDA, Malondialdehyde; Nrf2, nuclear factor erythroid 2-related factor 2; NQO-1, NAD(P)H Quinone Dehydrogenase 1; p38, p38 mitogen-activated protein kinase; p-p65, phosphorylated p65 transcription factor; p-ERK, phosphorylated extracellular signal-regulated kinase; p-JNK, phosphorylated-c-Jun N-terminal kinase; PTOA, post-traumatic osteoarthritis of ankle; SOD, superoxide dismutase; SIN, Sinomenine; SFFH, synovial fluid fracture hematoma; TRAF6, TNF-receptor associated factor-6; p-p38, phosphorylated p38; TNF-α, tumor necrosis factor.

## Data Availability

No new data were created or analyzed in this study. Data sharing is not applicable to this article.

## Abbreviations

IL-1RA: interleukin-1 receptor antagonist; MAPK, Mitogen-activated protein kinase; miR-146a, MicroRNA 146a; Nf-kB, nuclear factor kappa-light-chain-enhancer of activated B cells; NMP, N-methyl-2-pyrrolidone; Nrf-2, nuclear factor erythroid 2-related factor 2; RvD1, Resolvin D1; SIN, Sinomenine.
